# A comparison of the Charlson comorbidity index derived from medical records and claims data from patients undergoing lung cancer surgery in Korea: a population-based investigation

**DOI:** 10.1186/1472-6963-10-236

**Published:** 2010-08-13

**Authors:** Hyun-Ju Seo, Seok-Jun Yoon, Sang-Il Lee, Kun Sei Lee, Young Ho Yun, Eun-Jung Kim, In-Hwan Oh

**Affiliations:** 1 Department of Nursing, College of Medicine, Chosun University, Gwangju, Korea; 2 Department of Preventive Medicine, Korea University, Seoul, Korea; 3 Department of Preventive Medicine, University of Ulsan College of Medicine, Seoul, Korea; 4 Department of Preventive Medicine, Konkuk University School of Medicine, Seoul, Korea; 5 National Cancer Control Research Institute and Hospital, National Cancer Center, Seoul, Korea; 6 Graduate School of Korea University, Department of Public Health, Seoul, Korea

## Abstract

**Background:**

Calculating the Charlson comorbidity index (CCI) from medical records is a time-consuming and expensive process. The objectives of this study are to 1) measure agreement between medical record and claims data for CCI in lung cancer patients and 2) predict health outcomes of lung cancer patients based on CCIs from both data sources.

**Methods:**

We studied 392 patients who underwent surgery for pathologic stages I-III of lung cancer. The kappa value was used to measure the agreement between the 17 comorbidities of the CCI prevalence obtained from medical records and claims data. Multiple linear regression analyses were used to evaluate the relationships between CCI and length of stay and reimbursement cost.

**Results:**

Out of 17 comorbidities identified in the Charlson comorbidity index, ten had a higher prevalence, four had a lower prevalence and three had a similar prevalence in claims data to those of medical records. The kappa values calculated from the two databases ranged from 0.093 to 0.473 for nine comorbidities. In predicting length of stay and reimbursement cost after surgical resection for lung cancer patients, the CCI scores derived from both the medical records and claims data were not statistically significant.

**Conclusions:**

Poor agreement between medical record data and claims data may result from different motivations for collecting data. Further studies are needed to determine an appropriate method for predicting health outcomes based on these data sources.

## Background

Risk adjustment in clinical and health services research studies has been advocated because the effects of confounding factors in nonrandomized studies can be removed or reduced by adjusting the outcome measures according to risk [1]. The severity and comorbidities can be assessed to remove any potential causative factors for the observed variations in health outcomes between groups in order to differentiate treatment effects [2].

The Charlson comorbidity index (CCI) consists of 19 comorbid conditions weighted according to the degree to which they predict mortality among an inpatient cohort, and then the scores are summed to produce an index score [3,4]. The Charlson comorbidity index was developed based solely on the assessment of medical records. However, despite the widespread use of medical records, there is an inherent concern that there is a selection bias in the results. The differences in the type and number of comorbidities can result from the number of physician visits and the number of hospitalizations rather than an actual difference in comorbidities. In addition, a retrospective analysis of medical records would require great time investment and expense for a large cohort study. Therefore, an alternative data resource must contain the necessary information regarding comorbidities, and it must enable the performance of data collection and analysis in an efficient manner [5].

Fortunately, the method of calculating scores for CCI is easier than are the methods for other comorbidity indices. Therefore, medical records, patient reports, and claims databases are all potential data sources, though investigators must evaluate the quality, availability, and cost of each [6]. Claims-based measures of comorbidity are of particular importance to cancer care researchers, who increasingly use population-based cancer registry data linked with administrative claims to examine such issues as the associations among treatment, health expenditure, and survival [7].

The situation is similar in Korea. Based on the current privacy laws of Korea, access to medical records has been restricted for researchers. Though Korea has its own cancer registry, due to an insufficient amount of overall clinical information such as the comorbidities, use of the cancer registry data alone is not appropriate. As a result, attempts have been made to develop methods for adjusting the risks using the claims data. Though accuracy of disease coding has been questioned given the purpose of claims data, it could be a useful data source owing to its coverage [8]. Especially in Korea, more than 96% of the total Korean population is covered by national health insurance, and all national health insurance claims are reviewed by the Health Insurance Review & Assessment Service (HIRA), claims data of HIRA provides a fair representation of entire patient groups in Korea [9,10].

In this study, we compared the CCIs derived from medical chart review versus those based on claims data, and we examined whether the CCI scores obtained from the two data sources could predict the length of stay and the medical expenses, particularly for reimbursement cost by HIRA, in patients who underwent surgery for lung cancer.

## Methods

### Patients and CCI scoring method

Inpatient and outpatient medical records of patients who were initially diagnosed with lung cancer during a period ranging from January 1, 2000 to December 31, 2004 at the National Cancer Center were examined. We primarily selected patients who were preoperatively diagnosed with stage I to III disease, underwent surgery, and were 18 years of age or older. From 461 patients, 69 patients were excluded from the statistical analysis, because cancer stage or surgical treatment types of 64 patients were ill defined and five patients were diagnosed as state IV after the operation.

The final number of patients included in the study was 392, and the Charlson comorbidity conditions were collected using the Korean version of the CCI [11]. Additional clinical data collected from these patients included age, gender, pathologic stage, histologic type, surgical treatment modalities, frequency of surgery, surgical methods, date of operation, date of initial diagnosis of lung cancer and date of discharge. Comorbidities were considered to be present in cases in which any condition was confirmed more than once in the outpatient or inpatient medical records up to two years prior to the diagnosis of lung cancer. The medical records were analyzed by medical doctors in the Department of Family Medicine at the National Cancer Center from November 2007 to February 2008.

To calculate CCI values from the claims data, we collected the outpatient and hospitalization data for the same subjects using the Electronic Data Interchange (EDI) request data between 1999 and 2005. Information such as the date of treatment initiation, primary and secondary diagnosis codes, and reimbursement cost were gathered from the claims data of 392 subjects.

ICD-10 codes are used in Korea. Therefore, definitions of 17 comorbidities were adopted using an ICD-10 algorithm for CCI, developed by Quan et al. [12]. Based on the CCIs calculated using the claims data, comorbidities were determined according to ICD-10 codes. By assigning the weighted CCI values to the corresponding diseases, the sums of the scores were determined to be the final CCI scores.

We examined the EDI data from the two years preceding the initial date of confirmation of lung cancer codes [C33-C34] at the National Cancer Center, and then we confirmed the presence of comorbidity as defined by Charlson comorbidity conditions claimed to the HIRA for primary or secondary diagnoses. The EDI data were collected from all of the medical institutions in the country including the National Cancer Center. To enhance the diagnostic accuracy of comorbidities in claims data, a rule-out algorithm proposed by Klabunde et al. was applied [6]. This algorithm states that any condition confirmed more than twice in the data that was collected during the same period is considered to be comorbidity [6]. This study was approved by the Institutional Review Board of the National Cancer Center.

### Outcome definition

To estimate the reimbursement costs that were requested between the date of operation and the date of discharge, we summed the costs requested within a one-month period after the date of discharge at the National Cancer Center. Cost was transformed into a logarithm for analysis because of the right-skewed distribution of cost. The length of stay was considered to be the date of operation to that of discharge [13]. The length of stay also had a right-skewed distribution and was converted into a normal distribution via logarithmic transformation. In a model assessing the predictive validities of medical outcomes, CCI scores based on the medical records and claim data, which were obtained for each individual patient, were categorized according to three scales: 0, 1 and 2 or greater. These CCI scales were selected as the independent variables for health outcomes. Also we considered age, gender, histologic types of cancer, surgical treatment modalities and pathologic disease stages (stage I, II, III) as adjustable variables, as they have been reported to be prognostic factors of lung cancer in previous studies [14-16].

### Statistical analysis

The agreement between medical records and claims data on CCI was assessed based on simple agreement rate and kappa statistics. We performed a multiple linear regression analysis to evaluate whether CCI could predict length of stay and reimbursement cost [17]. All statistical analyses were performed using the Stata/SE 9.0 software package, and statistical significance was tested at a value of p < 0.05.

## Results

### Agreement between medical records and administrative data

The prevalence of 17 comorbidities and the agreement rate between the two data resources were analyzed from CCIs based on medical records and claims data, and the results are presented in Table 1.

**Table 1:** Agreements between medical records and claims data (unit = n (%))

Comorbidity	Prevalence medical records data	Prevalence administrative data	Agreement between data sources (Medical record/Administrative data)				Kappa(SE)	Agreement rate
			Yes/Yes	No/No	Yes/No	No/Yes		
Myocardial infarction	4(1.0)	5(1.3)	1(0.3)	384(98.0)	3(0.8)	4(1.0)	0.213(0.050)	98.21
Congestive heart failure	1(0.3)	9(2.3)	0(0.0)	382(97.4)	1(0.3)	9(2.3)	NA^c^	97.45
Peripheral vascular disease	1(0.3)	11(2.8)	0(0.0)	380(96.9)	1(0.3)	11(2.8)	NA^c^	96.94
Cerebrovascular disease	23(5.9)	21(5.4)	11(2.8)	359(91.5)	12(3.1)	10(2.6)	0.473(0.050)	94.39
Dementia	0(0.0)	0(0.0)	0(0.0)	392(100.0)	0(0.0)	0(0.0)	NA^b^	100.00
Chronic pulmonary disease	29(7.4)	138(35.2)	17(4.3)	242(61.7)	12(2.1)	121(30.9)	0.093(0.034)	66.07
Rheumatic disease	3(0.8)	10(2.6)	1(0.3)	380(96.9)	2(0.5)	9(2.3)	0.144(0.042)	97.19
Peptic ulcer disease	13(3.3)	91(23.2)	4(1.0)	292(74.5)	9(2.3)	87(22.1)	0.020(0.031)	75.51
Mild liver disease	9(2.3)	43(11.7)	6(1.5)	343(87.5)	3(0.8)	40(10.2)	0.187(0.036)	89.03
Diabetes without chronic complication	53(13.5)	15(3.8)	11(2.8)	335(85.5)	42(10.7)	4(1.0)	0.281(0.041)	88.27
Diabetes with chronic complication	6(1.5)	21(5.4)	3(0.8)	368(93.9)	3(0.8)	18(4.5)	0.203(0.041)	94.64
Hemiplegia or paraplegia	1(0.3)	1(0.3)	0(0.0)	390(99.5)	1(0.3)	1(0.3)	NA^c^	99.49
Renal disease	2(0.5)	0(0.0)	0(0.0)	390(99.5)	2(0.5)	0(0.0)	NA^a^	99.49
Any malignancy, except malignant neoplasm of the skin	10(2.6)	11(2.8)	4(1.0)	375(95.7)	6(1.5)	7(1.8)	0.364(0.050)	96.68
Moderate or severe liver disease	2(0.5)	1(0.3)	0(0.0)	389(99.2)	2(0.5)	1(0.3)	NA^c^	99.23
Metastatic solid tumor	0(0.0)	1(0.3)	0(0.0)	391(99.7)	0(0.0)	1(0.3)	NA^a^	99.74
AIDS/HIV	0(0.0)	0(0.0)	0(0.0)	392(100.0)	0(0.0)	0(0.0)	NA^b^	100.00

^a^Comorbidity was present in only one source; ^b^Comorbidity was absent in both sources; ^c^The cell frequency is 0.

In cases of myocardial infarction, congestive heart failure, peripheral vascular diseases, chronic pulmonary disease, rheumatoid diseases, peptic ulcer diseases, mild hepatic diseases, complicated diabetes mellitus, and metastatic solid cancer, the comorbidities of claims data were more prevalent than were the medical records data. In cases of primary cancer excluding skin cancer, quadriplegia, and hemiplegia, there were similar frequencies between the two data resources. Moreover, the prevalence of dementia and AIDS were 0% in both datasets.

To assess the agreements for 17 comorbidities between the medical records and claims data, kappa statistics and agreement rate were used. To calculate the kappa value, none of the cell frequencies should be zero, although this was observed in eight comorbidities of our study. Hence, the kappa value was calculated based on nine comorbidities. The agreement rate ranged from 66.07% to 100% for each disease, with chronic pulmonary disease having the lowest agreement rate. The kappa analysis revealed that the kappa value of four diseases, myocardial infarction, cerebrovascular disease, uncomplicated diabetes mellitus, and any malignancy except skin neoplasm, illustrated to fair agreement. In the remaining five diseases, which consisted of chronic pulmonary disease, rheumatoid diseases, peptic ulcer diseases, mild hepatic diseases, and complicated diabetes mellitus, the kappa value showed poor agreement. The concordance rates of the CCI scores between the two data resources are shown in Table 2. Agreement for CCI scores calculated by assigning comorbidities with weighted values had a kappa value of 0.054 (standard error = 0.029). For CCI scores categorized into three scales (i.e., 0, 1, 2+), the kappa statistic was 0.096 (standard error = 0.032). This indicates that the agreement was slightly increased, although it was still poor. According to the distributions of CCI scores between the two data resources, we observed that 196 patients (50.0%) had higher CCI scores as determined using the claims data than for that calculated using the medical records, with 45 patients (11.5%) having lower scores.

**Table 2:** Agreement in Charlson comorbidity index score between medical records and claims data

Claims data CCI score	Medical records CCI score						Total
	0	1	2	3	4	5	
0	**122**	26	3	3	0	0	154
1	90	**22**	6	1	0	0	119
2	41	24	**5**	3	0	0	73
3	14	11	6	**2**	0	3	36
4	2	1	1	1	**0**	0	5
5	1	2	0	0	1	**0**	4
8	0	0	0	1	0	0	1
Total	270	86	21	11	1	3	392

Note: kappa = 0.054 (SE 0.029) (agreement rate = 38.52%). Bold characters represent a complete concordance of the frequency between the medical record and claims data.

### Subject characteristics

For 392 patients who underwent surgery for lung cancer, the mean age was 60.9 years (standard deviation = 8.8 years). The number of male patients was 291 (74.2%), accounting for the majority of patients (Table 3).

**Table 3:** Patient characteristics and operative characteristics (n = 392)

Characteristics		n	%
Age[year]	≤ 49	46	11.7
	50-59	108	27.6
	60-69	169	43.1
	≥ 70	67	17.1
	Mean ± SD	60.9 ± 8.8	
Gender	male	291	74.2
	female	101	25.8
One-year mortality	alive	384	98.0
	dead	8	2.0
Three-year mortality	alive	358	91.3
	dead	34	8.7
Surgical treatment type	wedge resection	12	3.1
	lobectomy	336	85.7
	pneumonectomy	41	10.5
	other	3	0.8
Pathologic stage	I	206	52.6
	II	76	19.4
	III	110	28.0
Histologic subtype^a^	squamous cell carcinoma	204	53.0
	adenocarcinoma	155	40.3
	bronchoalveolar carcinoma	17	4.4
	large cell carcinoma	2	0.5
	small cell carcinoma	3	0.8
	other	4	1.0
Length of stay (days)	Mean ± SD	37.60 ± 46.32	
Mean reimbursement cost(won)	Mean ± SD	5,240,683 ± 2,318,995	

^a^Seven patients were missing histologic subtype.

### Prediction for length of stay

For the length of stay prediction adjusted by age, gender, histologic differentiation of the cancer, surgical treatment modalities, and pathologic stage, the CCI scores based on medical records and claims data are shown in Table 4. The CCI scores derived from either database were not prognostic for length of stay after adjusting for age, gender, histologic differentiation of the cancer, surgical treatment modalities, and pathologic stage. However, length of stay significantly increased by 1.15 times (95% CI 1.005-1.314) with pathologic stage 3 lung cancer in the claims data model.

**Table 4:** Prediction for length of stay using medical records and claims data-based comorbidity index score

		Exp[coefficient] based on medical records	95% Confidence interval	Exp[coefficient] based on claims data	95% Confidence interval
Age[years]	≤ 49	1		1	
	50-59	1.06	(0.880-1.295)	1.06	(0.871-1.282)
	60-69	1.2	(1.000-1.448)	1.19	(0.986-1.427)
	≥ 70	1.07	(0.866-1.328)	1.05	(0.846-1.305)
Gender	male	1		1	
	female	0.96	(0.832-1.113)	0.96	(0.833-1.116)
Histologic subtype	squamous cell carcinoma	1		1	
	adenocarcinoma	0.95	(0.834-1.094)	0.96	(0.839-1.101)
	bronchoalveolar carcinoma	1.1	(0.827-1.471)	1.1	(0.827-1.473)
	large cell carcinoma	0.79	(0.372-1.675)	0.8	(0.377-1.709)
	small cell carcinoma	1.21	(0.654-2.268)	1.19	(0.640-2.227)
	other	0.94	(0.552-1.626)	0.96	(0.558-1.639)
Surgical treatment type	wedge resection	1		1	
	lobectomy	1.15	(0.832-1.607)	1.18	(0.851-1.641)
	pneumonectomy	0.98	(0.674-1.438)	1.01	(0.688-1.469)
	other	1.43	(0.721-2.864)	1.46	(0.729-2.913)
Pathologic stage	I	1		1	
	II	0.96	(0.832-1.123)	0.97	(0.838-1.132)
	III	1.13	(0.993-1.302)	1.15*	(1.005-1.314)
CCI score	0	1		1	
	1	0.92	(0.809-1.062)	0.99	(0.863-1.128)
	2+	0.94	(0.775-1.144)	1	(0.872-1.142)

*p < 0.05 according to multiple linear regression analysis using log-transformed length of stay

### Prediction for reimbursement cost

Based on the medical records and claims data, a multiple linear regression analysis was performed to examine the predictive power of CCI-based reimbursement cost. After adjusting for age, gender, histologic differentiation of the cancer, surgical treatment modalities, and pathologic stage, CCI scores were not selected as a prognostic factor. The only reimbursement cost was significantly higher at pathologic stage 3 of lung cancer by 1.15 times in claims data based model (95% CI 1.020-1.295) (Table 5).

**Table 5:** Prediction for reimbursement cost using medical records and claims data-based comorbidity index score

		Exp[coefficient] based on medical records	(95% Confidence interval)	Exp[coefficient] based on claims data	(95% Confidence interval)
Age[years]	≤ 49	1		1	
	50-59	1	(0.842-1.187)	1.02	(0.859-1.213)
	60-69	0.92	(0.787-1.093)	0.96	(0.813-1.131)
	≥ 70	0.87	(0.727-1.063)	0.91	(0.751-1.106)
Gender	male	1		1	
	female	0.96	(0.852-1.104)	0.97	(0.849-1.102)
Histologic subtype	squamous cell carcinoma	1		1	
	adenocarcinoma	0.98	(0.872-1.110)	0.97	(0.863-1.100)
	bronchoalveolar carcinoma	0.69	(0.535-0.893)	0.69	(0.530-0.889)
	large cell carcinoma	1.05	(0.540-2.058)	1	(0.508-1.964)
	small cell carcinoma	0.38	(0.219-0.663)	0.39	(0.226-0.688)
	other	0.84	(0.521-1.362)	0.84	(0.517-1.354)
Surgical treatment type	wedge resection	1		1	
	lobectomy	0.96	(0.721-1.296)	0.93	(0.697-1.253)
	pneumonectomy	1.02	(0.733-1.438)	1.01	(0.720-1.417)
	others	0.78	(0.425-1.451)	0.75	(0.402-1.386)
Pathologic stage	I	1		1	
	II	1.05	(0.923-1.206)	1.04	(0.908-1.188)
	III	1.17*	(1.044-1.328)	1.15*	(1.020-1.295)
CCI score	0	1		1	
	1	1.11	(0.986-1.256)	0.98	(0.870-1.104)
	2+	1.15	(0.972-1.374)	0.97	(0.859-1.094)

*p < 0.05 according to multiple linear regression analysis using log-transformed reimbursement cost

## Discussion

CCI was originally developed based on data derived from medical records. However, many researchers have proposed that the sole dependence on medical records results in a limitation in cases for which a prompt risk assessment is required by hospitals, insurers and Health Management Organizations (HMOs) [18]. Therefore, CCI tools have been developed for use with claims data coded using ICD-9-CM (International Classification of Diseases, 9th revision, Clinical Modification) [19,20] and ICD-10 (Switzerland, Australia, Canada versions) [12,21,22].

However, the consistency between medical records and claims data is not excellent. For 485 patients who underwent prostatectomy, the comorbidities based on the medical records and claims data were compared. The kappa value was greater than 0.61 for five diseases (uncomplicated diabetes mellitus, primary solid tumor, moderate hepatic disease, connective tissue diseases, leukemia), but the remaining diseases had poor agreement [23]. Also Newschaffer et al. compared CCI scores between the medical records and claims data in 404 patients with breast cancer. This comparison revealed that the kappa value was 0.36, corresponding to 'fair agreement' [24].

In a Korean study that targeted patients who underwent surgery for the treatment of gastric cancer, the kappa value of CCI comorbidities between the data resources was fair for five diseases; however, excluding these comorbidities, the kappa value was less than 0.2. Especially the prevalence of peptic ulcer disease was 41.6% according to the claims data and 3.5% according to the medical records data [25]. Another study, which focused on patients who underwent hip joint arthroplasty, reported that the kappa value of comorbidities for two data resources was 0.8 for metastatic solid tumors and 0.51 for uncomplicated diabetes mellitus. In other comorbidities, the kappa value was smaller than 0.29 [26]. In this study, there were discrepancies between the two data sources, supporting the results from previous studies in Korea and other countries.

One possible explanation for this disagreement is that there is an underestimation of CCI comorbidities based on medical records data. Medical records based on CCI scores were retrospectively obtained from the one medical institution, whereas claims data based on CCI scores were gathered from administrative data, which includes all medical institutions' claims data on the selected patient. In addition, poor agreement between medical record data and claims data may result from the differing motivations for data collection between medical records and claims data.

Inconsistencies have also been observed in previous studies investigating whether CCI scores obtained from claims data could be used to predict the length of hospital stay. According to a study of 20,138 patients who underwent surgery for radical urological cancer, the length of stay was prolonged by approximately 1-2 days in the group in whom CCI scores were at least 1 compared to the groups with 0 points [27]. In another study, 1,216 patients who visited an outpatient clinic with a chief complaint of acute chest pain, compared with the group in which CCI scores were calculated to be 0-1 points from the medical records, the length of stay was delayed by 14.4-fold (95%CI 3.9-25.9) in the group in which CCI scores were calculated to be 2-3 points and by 25.3-fold (95%CI 2.4-25.5) in the group in which CCI scores were calculated to be < 4 points [28]. However, in 1,945 patients who underwent carotid endarterectomy, CCI scores based on claims data did not correlate with the length of stay, though CCI scores based on medical records were associated with an increased length of stay [1]. In the current study, according to a length of stay prediction model, CCI scores calculated using medical records and claims data were not shown to be prognostic factors. This may be due to the fact that CCI was originally developed to predict mortality, and it is simply not an appropriate tool for predicting length of stay [29]. Also, the postoperative length of stay may be dependent on the severity of the procedure rather than comorbidities [27].

Moreover, there were disagreements in the predictability of CCI with regard to reimbursement cost. In a medical record-based study of dialysis patients, the mean medical expense per year was $54,000 in cases in which CCI scores were calculated to be less than 4 points, $108,000 in those where CCI scores were calculated to be 4-5 points, $247,000 in those where CCI scores were calculated to be 6-7 points and $407,000 in those where CCI scores were calculated to be 8 or more points. These differences were found to be statistically significant (p < 0.0001) [30]. However, CCI scores did not correlate with medical expense in head-and-neck cancer patients [31]. In our study, according to a reimbursement cost prediction model that was established based on the medical records and claims data as related by CCI, CCI was not selected as a prognostic factor for either model. This could be attributed to the fact that the prediction model was developed for death hazards [29].

This study has several limitations. First, medical records based on the CCI scores of selected patients were retrospectively obtained from the records of the National Cancer Center. However, claims data based on the CCI scores of selected patients were gathered from HIRA, which includes all medical institutions' claims data on the selected patients. But this could be regarded as strength of claims data in terms of accessibility and efficiency. Second, we considered the reimbursement cost of claims data only with respect to the availability of data. As a result, other medical expenses, such as non-covered services, were excluded. Also, the reimbursement cost was calculated as the sum of the medical costs from the date of surgery to one month after the date of discharge. Therefore, it is possible that outpatient visits except follow-up cancer treatment within a one-month period after discharge may have been included. Also though we considered some prognostic factor such as pathologic staging, there are other prognostic factors such as smoking or adjuvant chemotherapy. More information about such factor could affect the health outcomes and the predictability of CCI.

In this study, there was poor agreement between medical records and claims data. In addition, CCIs based on both data sets were not suitable for predicting length of stay or medical expenses. Given how easy it is to calculate CCIs based on claims data, especially in a social health insurance system, there should be further studies to improve methods for calculating CCI scores to predict health outcome.

## Conclusions

Considering the difficulty of accessing medical records in the presence of privacy protection laws, the use of claims data to calculate CCI is a very attractive and potentially useful method. Claims data can also be used to supplement the medical records because claims data can be collected easily by different medical institutions. However, in this study, the agreement of CCIs based on the two datasets was not sufficient, and CCIs from neither data set were able to predict length of stay or medical expenses. Therefore, improvement and modification are needed to successfully use CCI scores based on claims data, at least in the case of lung cancer.

## Competing interests

The authors declare that they have no competing interests.

## Authors' contributions

SJY planned the design and carried out the critical revision of the manuscript and the supervision of all other parts of this study. HJS extracted the data and prepared a draft. SIL and KSL participated in the study design and contributed to critical revision of the manuscript. YHS extracted the data and performed statistical analysis. EJK and IHO summarized the data and contributed to the final version of the manuscript. All authors read and approved the final manuscript.

## Pre-publication history

The pre-publication history for this paper can be accessed here:

http://www.biomedcentral.com/1472-6963/10/236/prepub
